# Renal-Limited Presentation of Hydralazine-Induced Anti-neutrophil Cytoplasmic Antibody Vasculitis: A Case Report

**DOI:** 10.7759/cureus.69330

**Published:** 2024-09-13

**Authors:** Andrew Lurie, Lohitha Guntupalli, Nancy A Finnigan, Umair Ahmed

**Affiliations:** 1 Internal Medicine, Nova Southeastern University Dr. Kiran C. Patel College of Osteopathic Medicine, Clearwater, USA; 2 Osteopathic Medicine, Nova Southeastern University Dr. Kiran C. Patel College of Osteopathic Medicine, Clearwater, USA; 3 Medical Education, Lakeland Regional Health, Lakeland, USA; 4 Nephrology, Lakeland Regional Health, Lakeland, USA

**Keywords:** anca associated vasculitis, drug induced vasculitis, drug-related side effects, hydralazine associated vasculitis, hydralazine-induced vasculitis

## Abstract

Anti-neutrophil cytoplasmic antibody (ANCA)-associated vasculitis is a rare multisystem autoimmune disease resulting from necrotizing inflammation of small vessels. Genetic predisposition and environmental factors are typically associated with its presentation, though rarely a drug-induced form has been reported. Here, we present a case of a 73-year-old female with a history of hypertension and chronic kidney disease who presented with acute kidney injury secondary to hydralazine-induced ANCA vasculitis. This report aims to highlight the rare association of hydralazine with vasculitis and the importance of pursuing a full initial workup in patients with acute kidney injury.

## Introduction

Anti-neutrophil cytoplasmic antibody (ANCA)-associated vasculitis is a rare autoimmune disorder characterized histologically by necrotizing inflammation of small vessels. Evaluation of neutrophils with indirect immunofluorescence reveals two main immunofluorescence staining patterns: cytoplasmic (C-ANCA) and perinuclear (P-ANCA). C-ANCA is mainly directed against proteinase-3 while P-ANCA is against myeloperoxidase (MPO) [1]. The end result in either case is the induction of neutrophil overactivation and vessel injury [2]. Given the diffuse presence of small vessels, the spectrum of clinical presentation is broad and can vary from skin rash to multisystem disease. While any tissue may be affected, the most commonly damaged organs include the lower respiratory tract and the kidneys. There are three main histological forms of ANCA-associated vasculitis, each with distinct clinical phenotypes: granulomatosis with polyangiitis, microscopic polyangiitis, and eosinophilic granulomatosis with polyangiitis [3]. Most cases of ANCA vasculitis are thought to be driven by a combination of genetics and environmental factors [4], though rarely drug-induced ANCA-associated vasculitis is reported [5]. Hydralazine, a direct vasodilator, is most commonly implicated in drug-induced lupus, a syndrome consisting of clinical features such as fatigue, arthralgia, myalgia, and serositis resembling systemic lupus erythematosus. Less commonly, hydralazine can induce ANCA vasculitis. While the direct pathogenesis remains unknown, theories include autoantibody production secondary to hydralazine-MPO-induced neutrophil apoptosis and hydralazine-induced epigenetic silencing resulting in increased neutrophil auto-antigen expression [6]. Given the paucity of data and varied clinical presentation, drug-induced ANCA-associated vasculitis poses a diagnostic challenge leading to significant delay in treatment. A careful systematic approach and full diagnostic workup in patients developing multisystem involvement are critical in early detection and treatment.

## Case presentation

A 73-year-old female with a past medical history of hypertension and stage 3 chronic kidney disease with a baseline creatinine of 1.0 mg/dL presented to the Emergency Department for complaints of persistent weakness and fatigue for the past two weeks.

Initial laboratory workup (Table 1) revealed a serum hemoglobin of 6.8 mg/dL, blood urea nitrogen (BUN) of 49 mg/dL, and a serum creatinine of 5.4 mg/dL. Urinalysis (Table 2) revealed microscopic hematuria and subnephrotic range proteinuria. Initial management consisted of hemodynamic stabilization with transfusion of two units of packed red blood cells after which hemoglobin stabilized at 9.4 mg/dL. An esophagogastroduodenoscopy was performed and discovered a superficial nonbleeding duodenal ulcer. Due to worsening renal function after appropriate resuscitation, an alternative explanation was explored for the etiology of the acute kidney injury (AKI). The rapid nature of this patient's deterioration in the setting of urine sediment with microscopic hematuria raised concern for acute glomerulonephritis. Additional workup was pursued (Table 3), which was positive for antinuclear antibody (ANA), anti-histone and anti-MPO antibodies, and P-ANCA.

**Table 1:** Laboratory values on admission

Laboratory parameter	Value (reference range)	Unit
White blood cell count	8.2 (4.5-11.0) × 10^3^	Cells/microliter
Red blood cell count	2.62 (4.20-5.40) × 10^6^	Cells/microliter
Hemoglobin	6.8 (12.0-16.0)	g/dL
Hematocrit	21.4 (37.0-47.0)	%
Platelets	417 (150-450) × 10^9^	Cells per microliter
Sodium	139 (135-145)	mmol/L
Potassium	4.7 (3.5-5.3)	mmol/L
Chloride	104 (98-107)	mmol/L
Carbon dioxide	21 (21-32)	mmol/L
Blood urea nitrogen	76 (7-18)	mg/dL
Creatinine	5.4 (0.6-1.0)	mg/dL
Glucose	108 (70-100)	mg/dL

**Table 2:** Urine studies on admission

Laboratory parameter	Value (reference range)	Unit
Glucose	Trace (negative)	None
Bilirubin	Negative (negative)	None
Ketones	Negative (negative)	None
Specific gravity	1.011 (1.007-1.025)	None
Blood	3+ (negative)	None
pH	6.5 (4.5-8)	None
Protein	1+ (negative)	None
Nitrite	Negative (negative)	None
Leukocyte esterase	Negative (negative)	None
Microscopic red blood cells	Too numerous to count (negative)	Cells
White blood cells	Negative (negative)	Cells
Bacteria	Negative (negative)	Cells
Epithelial cell	Negative (negative)	Cells

**Table 3:** Serology workup for acute glomerulonephritis P-ANCA, perinuclear anti-neutrophil cytoplasmic antibody.

Laboratory parameter	Value (reference range)	Unit
Albumin	3.5 (3.8-4.8)	g/L
Alpha 1 globulin	0.6 (0.2-0.3)	g/L
Alpha 2 globulin	1.2 (0.5-0.9)	g/L
Beta globulin 1	0.6 (0.4-0.6)	g/L
Beta globulin 2	2 (0.2-0.5)	g/L
Gamma globulin	2 (0.8-1.7)	g/L
Abnormal bands	None (none)	None
Total protein	8.4 (6.1-8.1)	g/L
Glycated hemoglobin	5.5 (4.0-6.0)	%
Hepatitis B surface antigen	Non-reactive (non-reactive)	None
Hepatitis B surface antibody	Non-reactive (non-reactive)	None
Hepatitis B core antibody	Non-reactive (non-reactive)	None
Antinuclear antibody	1:1280 (negative)	None
Anti-DNA antibody	Negative (negative)	None
P-ANCA	>1:640 (<1:20)	None
Myeloperoxidase antibody	24.8 (negative)	Unit/mL
Proteinase-3 antibody	<1.0 (<1.0)	None
Complement C3	133 (90-180)	mg/dL
Complement C4	21.4 (10-40)	mg/dL

Given these acute changes and a medication history including hydralazine, a renal biopsy was sought for tissue diagnosis to better characterize the underlying disease. The biopsy revealed pauci-immune focal segmental crescenteric and necrotizing glomerulonephritis consistent with drug-induced vasculitis, and in this clinical setting suggested hydralazine-induced ANCA-associated vasculitis.

**Figure 1:**
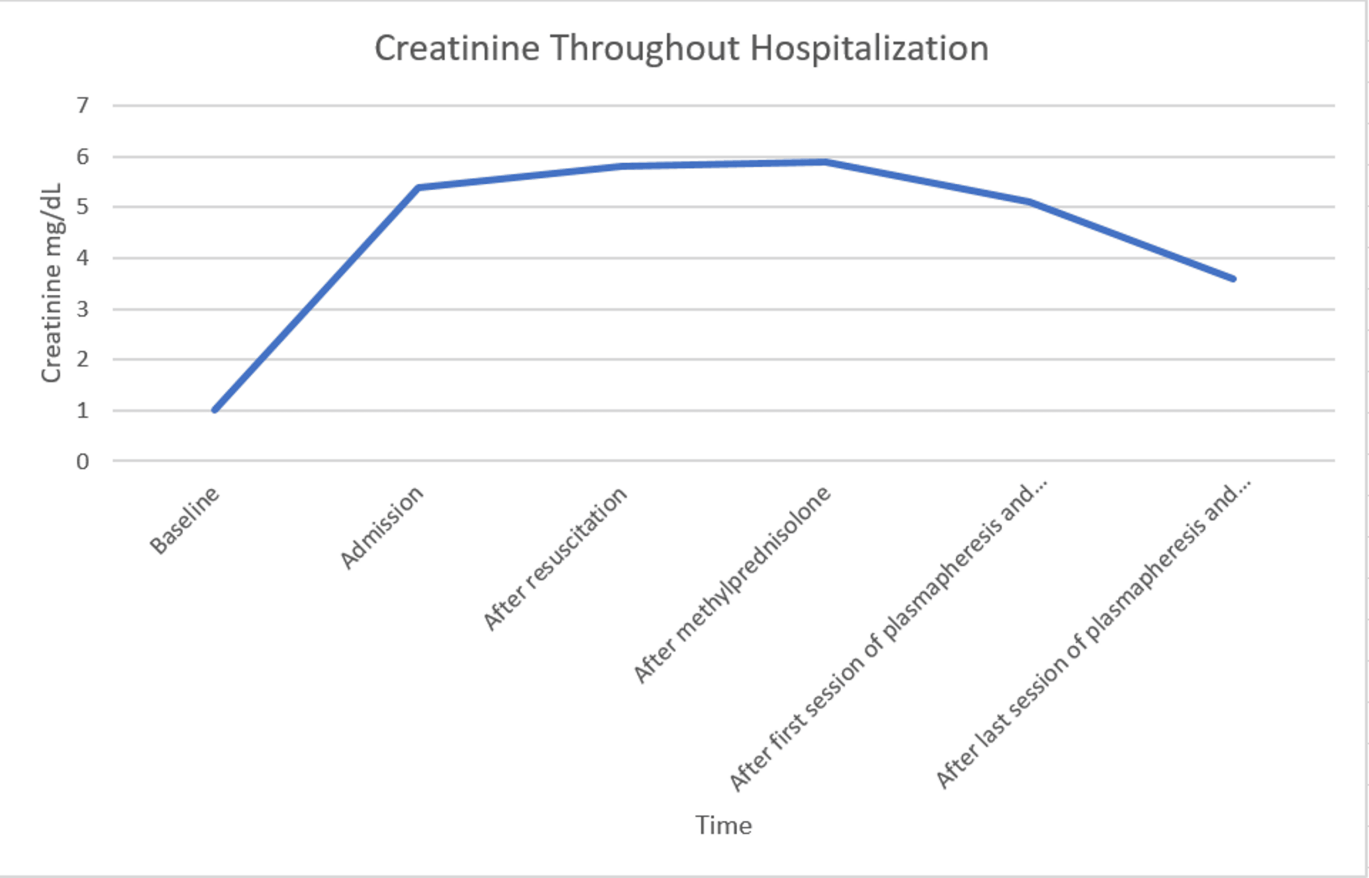
Creatinine throughout admission

Management consisted of discontinuing hydralazine and administering methylprednisolone 1 g daily intravenously for three days. Renal function remained stable during this period. Intravenous steroids were transitioned to an oral regimen starting at prednisone 60 mg daily and tapered over the next several weeks while more intensive management was continued. She was started on seven sessions of one-volume plasmapheresis replaced with fresh frozen plasma scheduled every other day. Concurrently rituximab 1 g was administered on day 1 of plasmapheresis followed by a second dose 14 days later. During this intervention, renal function improved significantly (Figure 1). After completing this therapy the patient was discharged home in stable condition.

At the most recent follow-up 15 months later, the patient has continued to improve. Her new baseline serum creatinine is 2.26 g/dL with no proteinuria or hematuria.

## Discussion

ANCA-associated vasculitis is a rare disease characterized by necrotizing inflammation of small vessels without immune complex deposition. Over the past 10 years, the amount of case reports reflecting patients with ANCA-associated vasculitis has slowly increased in number, though the annual incidence of these primary systemic vasculidities has been estimated at 19.8 cases per million [7]. Patients often present with nonspecific constitutional symptoms including fatigue, arthralgias, and fever. More severe cases cause organ-specific pathologies, with the most commonly affected tissues being the lower respiratory tract and kidneys. Case reports have shown patients to present most commonly with a pulmonary-renal syndrome, distinct from this case of a renal-limited variety.

Hydralazine is a direct arteriolar vasodilator commonly used in hypertensive emergencies, pregnancy, or as a second- or third-line drug in the management of chronic hypertension. Chronic hydralazine use is known to be associated with autoimmunity in the form of drug-induced lupus [8] but more recently, reports have revealed a link with drug-induced ANCA vasculitis [6]. Differentiating idiopathic and drug-induced ANCA vasculitis can be challenging. All patients with ANCA vasculitis tend to have positive ANCA antibodies; however, patients with drug-induced forms are often positive for ANA and anti-histone antibodies as well.

Management begins with prompt discontinuation of the offending medication, though this alone is often not sufficient. Further treatment modalities include the use of immunosuppressants -- commonly with rituximab or cyclophosphamide -- and possibly plasmapheresis for more severe organ involvement. Maintenance immunosuppressants are generally not needed once the offending drug is stopped as relapses are uncommon in these patients.

This case highlights the importance of urine studies in patients with AKI as unexplained microscopic hematuria could be suggestive of an underlying glomerulonephritis and warrant a nephrology evaluation [9]. Furthermore, it also supports the claim to avoid the use of hydralazine in favor of first- and second-line antihypertensive medications except in select populations. Hydralazine is one of the very few antihypertensives safe during pregnancy and has shown efficacy in lowering mortality in African American patients with heart failure with reduced ejection fraction when combined with isosorbide dinitrate [10]. Outside of these groups, hydralazine should be preferentially replaced with other agents when possible given its potential adverse effects. Drug-induced vasculitis can have catastrophic consequences and we recommend treating hypertension with first-line antihypertensives such as thiazide diuretics, angiotensin-converting enzyme (ACE) inhibitors or angiotensin II receptor blockers (ARBs), and dihydropyridine calcium-channel blockers.

## Conclusions

ANCA vasculitis is a devastating disease that can permanently alter renal function if left untreated. Presenting symptoms may be nonspecific with generalized weakness, fatigue, and arthralgias but quickly progresses to end-organ dysfunction, most commonly of the kidneys and lower respiratory tract. AKI has a broad differential. As such, care should be taken to order a urinalysis in every case of AKI in search of proteinuria, hematuria, and casts which would raise suspicion for a possible glomerulonephritis and require a deeper investigation. Although rare, hydralazine is developing an increasingly greater association with the development of ANCA vasculitis. We recommend preferentially using hydralazine in groups that have an absolute indication. Instead, we prefer first-line antihypertensives such as thiazide diuretics, ACE inhibitors or ARBs, and dihydropyridine calcium-channel blockers.

## References

[1] Radice A, Sinico RA (2005). Antineutrophil cytoplasmic antibodies (ANCA). Autoimmunity.

[2] Kitching AR, Anders HJ, Basu N (2020). ANCA-associated vasculitis. Nat Rev Dis Primers.

[3] Yates M, Watts R (2017). ANCA-associated vasculitis. Clin Med (Lond).

[4] Almaani S, Fussner LA, Brodsky S, Meara AS, Jayne D (2021). ANCA-associated vasculitis: An update. J Clin Med.

[5] Weng CH, Liu ZC (2019). Drug-induced anti-neutrophil cytoplasmic antibody-associated vasculitis. Chin Med J (Engl).

[6] Aeddula NR, Pathireddy S, Ansari A, Juran PJ (2018). Hydralazine-associated antineutrophil cytoplasmic antibody vasculitis with pulmonary-renal syndrome. BMJ Case Rep.

[7] Watts RA, Lane SE, Bentham G, Scott DG (2000). Epidemiology of systemic vasculitis: A ten-year study in the United Kingdom. Arthritis Rheum.

[8] Cameron HA, Ramsay LE (1984). The lupus syndrome induced by hydralazine: A common complication with low dose treatment. Br Med J (Clin Res Ed).

[9] Perazella MA, Coca SG, Kanbay M, Brewster UC, Parikh CR (2008). Diagnostic value of urine microscopy for differential diagnosis of acute kidney injury in hospitalized patients. Clin J Am Soc Nephrol.

[10] Ziaeian B, Fonarow GC, Heidenreich PA (2017). Clinical effectiveness of hydralazine-isosorbide dinitrate in African-American patients with heart failure. JACC Heart Fail.

